# Fructose-Bisphophate Aldolase Exhibits Functional Roles between Carbon Metabolism and the *hrp* System in Rice Pathogen *Xanthomonas oryzae* pv. *oryzicola*

**DOI:** 10.1371/journal.pone.0031855

**Published:** 2012-02-22

**Authors:** Wei Guo, Li-fang Zou, Yu-rong Li, Yi-ping Cui, Zhi-yuan Ji, Lu-lu Cai, Hua-song Zou, William C. Hutchins, Ching-hong Yang, Gong-you Chen

**Affiliations:** 1 Key Laboratory of Urban (South) by Ministry of Agriculture, School of Agriculture and Biology, Shanghai Jiao Tong University, Shanghai, China; 2 Key Laboratory of Integrated Management of Crop Diseases and Pests, Ministry of Education of China, College of Plant Protection, Nanjing Agricultural University, Nanjing, China; 3 Department of Biological Sciences, University of Wisconsin, Milwaukee, Wisconsin, United States of America; East Carolina University School of Medicine, United States of America

## Abstract

Fructose-bisphophate aldolase (FbaB), is an enzyme in glycolysis and gluconeogenesis in living organisms. The mutagenesis in a unique *fbaB* gene of *Xanthomonas oryzae* pv. *oryzicola*, the causal agent of rice bacterial leaf streak, led the pathogen not only unable to use pyruvate and malate for growth and delayed its growth when fructose was used as the sole carbon source, but also reduced extracellular polysaccharide (EPS) production and impaired bacterial virulence and growth in rice. Intriguingly, the *fbaB* promoter contains an imperfect PIP-box (plant-inducible promoter) (TTCGT-N_9_-TTCGT). The expression of *fbaB* was negatively regulated by a key *hrp* regulatory HrpG and HrpX cascade. Base substitution in the PIP-box altered the regulation of *fbaB* with the cascade. Furthermore, the expression of *fbaB* in *X. oryzae* pv. *oryzicola* RS105 strain was inducible *in planta* rather than in a nutrient-rich medium. Except other *hrp-hrc-hpa* genes, the expression of *hrpG* and *hrpX* was repressed and the transcripts of *hrcC*, *hrpE* and *hpa3* were enhanced when *fbaB* was deleted. The mutation in *hrcC*, *hrpE* or *hpa3* reduced the ability of the pathogen to acquire pyruvate and malate. In addition, bacterial virulence and growth *in planta* and EPS production in RΔ*fbaB* mutant were completely restored to the wild-type level by the presence of *fbaB in trans*. This is the first report to demonstrate that carbohydrates, assimilated by *X. oryzae* pv. *oryzicola*, play critical roles in coordinating *hrp* gene expression through a yet unknown regulator.

## Introduction

Carbohydrate nutrient acquisition is essential for bacterial pathogen growth to establish successful infections in host plants [1], [2], [3]. As in other living organisms, plant pathogenic bacteria carry out the catabolic process via the Emden-Meyerhof-Parnas (EMP) pathway of glycolysis, Entner–Doudoroff (ED), pentose phosphate pathway (PPP) and terminal oxidation mediated by the tricarboxylic acid (TCA) cycle to break down hexoses, like glucose, outside of their cells for energy and carbon molecules. Bacteria may also use gluconeogenesis to synthesize glucose from non-sugar C_2_ or C_3_ compounds or the intermediates of the TCA cycle when there is not sufficient hexoses in their immediate environment [4]. In *Xanthomonas*, ED, in conjunction with TCA, has been confirmed to be the predominant pathway for glucose catabolism, and a small portion (8 to 16%) of substrate glucose is routed into PPP [5], whereas the EMP pathway of glycolysis does not play a significant role in glucose catabolism, since *Xanthomonas* species, including rice bacterial leaf streak *X. oryzae* pv. *oryzicola*, lack an essential phosphofructokinase activity which converts fructose 6-phosphate to fructose-1,6-bisphosphate [5], [6], [7]. Moreover, little is known about the relationship of carbon metabolism to virulence.

The genome data of *X. oryzae* pv. *oryzicola* (http://cmr.jcvi.org/cgi-bin/CMR/GenomePage.cgi?org=Xoc), *X. oryzae* pv. *oryzae* [8], [9], *X. campestris* pv. *campestris* [10], *X. axonopodis* pv. *citri* [11] and *X. campestris* pv. *vesicatoria* [12] show that xanthomonads possess essential genes for the EMP pathway of glycolysis, ED, PPP, gluconeogenesis and TCA cycle. Currently, great interests have been focused on whether or not and how the carbon metabolic pathways are involved in the virulence of plant pathogenic bacteria. For example, the glyceraldehyde-3-phosphate dehydrogenase (GAPDH), converting glyceraldehyde 3-phosphate to 1,3-bisphosphoglycerate, is required for ED, extracellular polysaccharide (EPS) production and full virulence of *X. campestris* pv. *campestris* [13]. The phosphogluconate dehydratase gene (*edd*) in ED is necessary for xanthan biosynthesis and the 6-phosphogluconate dehydrogenase gene (*gndA*) in PPP does not influence xanthan biosynthesis in *X. oryzae* pv. *oryzae* [6]. The malate: quinone oxidoreductase gene (*mqo*) in TCA cycle is required for the wild-type growth, disease symptom development and full virulence of *Pseudomonas syringae* pv. *tomato* DC3000 in *Arabidopsis thaliana* [14]. The phosphoenolpyruvate synthase gene (*ppsA*), converting pyruvate to phosphoenolpyruvate, is essential for gluconeogenesis, *in planta* growth, and full virulence of *X. campestris* pv. *campestris* [4]. However, little is known about other carbon metabolic factors.

Previous reports have confirmed that some carbohydrates and sulfur-containing amino acids have the ability to induce the expression of *hrp* genes in Gram-negative phytopathogenic bacteria [15], [16], [17], [18]. The *hrp* genes, normally within a 25–27 kb gene cluster in *Xanthomonas* species, encoding a type-III secretion system (T3SS), enable bacterial pathogens to trigger a rapid, localized, programmed hypersensitive response (HR) in nonhost plants and become pathogenic in hosts [18], [19], [20]. Expression of *hrp* genes is actually suppressed in nutrient-rich media but induced *in planta* and in apoplast-mimicking media, XVM2 containing sucrose and fructose for *X. campestris* pathovars or species [17], [18], [21], [22], [23]; XOM3 only containing xylose for *X. oryzae* pathovars [20], [24], [25], except inorganic salt(s), implying that some nutrients released from plant tissues, which are degraded for bacterial growth, may have effects on induction of *hrp* gene expression. For instance, the *hrp* expression in *Ralstonia solanacearum* is activated possibly by ubiquitous and non-diffusible molecules in the presence of pathogen-plant cell contact [15], [16], [26]. The above prompts us to assume that there are unknown correlations between carbon metabolism and the *hrp* system for bacterial pathogenesis in plants.

When *Xanthomonas* species interact with plants, some of the *hrp* gene products generate a pedestal-like T3S structure that traverses the two bacterial membranes [27], [28]. For example, a pilus-like secretion channel (HrpE), which is outside of HrcC [29], and also a translocon protein (HrpF) in the plant membrane [27], [30], [31], [32], [33]. As a whole, the T3S apparatus injects a number of effectors into the apoplast and cytosol of plant cells leading to disease in hosts or HR in non-hosts. Conceptually, expression of the *hrp* genes is controlled by two key regulatory genes, *hrpG* and *hrpX*, which are located outside of the *hrp* gene cluster [19]. HrpG is predicted to be an OmpR-type response regulator of a two-component signal transduction system and presumably perceives an environmental signal via an unknown sensor kinase [34], [35]. HrpX is an AraC-type of transcriptional activator [36] which forms a homodimer containing a helix-turn-helix domain which interacts with each TTCGC motif of the PIP-box (plant-inducible promoter) in the *hrpB to hrpF* promoter regions to activate transcription of *hrp* [26], [37], [38], [39] and T3S effector genes [34], [35], [40]. The PIP-box has been taken to identify novel HrpX regulons which possess a PIP-box upstream of a 30–32 base pairs followed by a conserved -10 box-like sequence, as TTCGB-N_15–16_-TTCGB- N_30–32_-YANNNT (B refers to the base C, G, or T but not A) in the promoter region [36], [41], [42], [43]. But few genes, like *hrpF* with an imperfect PIP-box (TTCGC-N_8_-TTCGT) or without following the -10 box-like motif in the promoter region, have been described as being expressed in a HrpX-dependent manner [38], [39]. Recently, the coordinated expression of *Xanthomonas hrp-hrc-hpa* expression is orchestrated by multiple two-component systems and transcriptional regulators such as Trh [44], Clp [45], Zur [40], LrpX [46], ColR/S [47], and PhoP/Q [48]. However, the expression of *hrcC, hrpE and hpa3* genes is not obviously and completely controlled by these regulators, including HrpG and HrpX, in *X. oryzae* pv. *oryzicola* when the pathogen grows in *hrp*-inducing medium and *in planta* [20], implying that unknown regulator(s) may play roles in *hrp* gene expression.

To investigate uncertainty above, we screened our previous Tn5-tagged mutant library of *X. oryzae* pv. *oryzicola* [49] and got a mutant Mxoc0504 where the Tn5 was inserted in a unique gene *fbaB*. In this report, we present genetic evidence demonstrating that *fbaB* is required for gluconeogenesis, EPS production and the expression of *hrp* genes, as well as the full virulence of *X. oryzae* pv. *oryzicola* in rice.

## Materials and Methods

### Bacterial strains, culture media and growth conditions

Strains and plasmids used in this study are listed in Table 1. *Escherichia coli* strains were routinely grown in LB (Luria-Bertani) medium at 37°C [50]. *X. oryzae* pv. *oryzicola* strains were performed at 28°C in NA (1 g/L yest extract, 3 g/L beef extract, 5 g/L polypeptone, 10 g/L sucrose, 15 g/L agar), NB (NA without agar), NAN (NA without sucrose) or NAS (NA with 100 g/L sucrose), NY (NB without beef extract and sucrose), the non-carbohydrate minimal medium (NCM) (2 g/L (NH_4_)_2_SO_4_, 4 g/L K_2_HPO_4_, 6 g/L KH_2_PO_4_, 0.2 g/L MgSO_4_ · 7H_2_O) [4] or rice suepension cells [25] when required. Antibiotics were used when required at the following concentrations: kanamycin (Kan), 25 µg/ml; rifampicin (Rif), 50 µg/ml; ampicillin (Amp), 100 µg/ml and spectinomycin (Sp), 50 µg/ml.

**Table 1:** Strains and plasmids used in this study.

Strains or plasmids	Relevant characteristics^a^	Reference or source
*E. coli*		
DH5α	F^—^ Φ80d*lacZ* ΔM15Δ(*lacZYA-argF*)U169 *endA1 deoR recA1 hsdR17*(r_K_^—^ m_K_^+^) *phoA supE44 λ^—^ thi-l gyrA96 relA1*	Clontech
S17-1λpir	*recA*,*Thi*, *pro*, *hsdR^−^*, *M^+^*, *RP4: 2-Tc*::*Mu:KmTn7*, λpir, *Tp^r^*, *Sm^r^*	This lab
*X. oryzae* pv. *oryzicola*		
RS105	Wild type, the causal agent of bacterial leaf streak in rice, Rif^r^	This lab
RΔ*hrpG*	A *hrpG* knock-out mutant of strain RS105, Rif^r^	[53]
RΔ*hrpX*	A *hrpX* deletion mutant of strain RS105, Rif^r^	[53]
RΔ*hrcV*	A *hrcV* deletion mutant of strain RS105, Rif^r^	[65]
RΔ*hrpE*	A *hrpE* deletion mutant of strain RS105, Rif^r^	[65]
RΔ*hrcC*	A *hrcC* deletion mutant of strain RS105, Rif^r^	[20]
RΔ*hpa3*	A *hpa3* deletion mutant of strain RS105, Rif^r^	[20]
Mxoc0504	A *Xoryp_17640*::Tn5 inserted mutant, Rif^r^, Km^r^	This work
RΔ*fbaB*	A *fbaB* deletion mutant of strain RS105, Rif^r^	This work
CRΔ*fbaB*	RΔ*fbaB* harboring pCfbaB, Rif^r^, Sp^r^	This work
RS105(pfbaBaGUS)	The wild-type RS105 harboring pfbaBaGUS, Rif^r^, Sp^r^	This work
RS105(pfbaBbGUS)	The wild-type RS105 harboring pfbaBbGUS, Rif^r^, Sp^r^	This work
RS105(pfbaBcGUS)	The wild-type RS105 harboring pfbaBcGUS, Rif^r^, Sp^r^	This work
RS105(pfbaBdGUS)	The wild-type RS105 harboring pfbaBdGUS, Rif^r^, Sp^r^	This work
RΔ*hrpG*(pfbaBaGUS)	RΔ*hrpG* mutant harboring pfbaBaGUS, Rif^r^, Sp^r^	This work
RΔ*hrpG*(pfbaBbGUS)	RΔ*hrpG* mutant harboring pfbaBbGUS, Rif^r^, Sp^r^	This work
RΔ*hrpG*(pfbaBcGUS)	RΔ*hrpG* mutant harboring pfbaBcGUS, Rif^r^, Sp^r^	This work
RΔ*hrpG*(pfbaBdGUS)	RΔ*hrpG* mutant harboring pfbaBdGUS, Rif^r^, Sp^r^	This work
RΔ*hrpX*(pfbaBaGUS)	RΔ*hrpX* mutant harboring pfbaBaGUS, Rif^r^, Sp^r^	This work
RΔ*hrpX*(pfbaBbGUS)	RΔ*hrpX* mutant harboring pfbaBbGUS, Rif^r^, Sp^r^	This work
RΔ*hrpX*(pfbaBcGUS)	RΔ*hrpX* mutant harboring pfbaBcGUS, Rif^r^, Sp^r^	This work
RΔ*hrpX*(pfbaBdGUS)	RΔ*hrpX* mutant harboring pfbaBdGUS, Rif^r^, Sp^r^	This work
**Plasmids**		
pMD18-T	pUC *ori*, cloning vector, Ap^r^	Takara
pKMS1	Suicide vector derivative from pK18mobGII, sacB^+^, Km^r^	This lab
pHM1	Sp^r^ or Sm^r^ *IncW*, *Mob(p), Mob^+^, LacIP^+^*, PK2 replicon, cosmid	This lab
pKΔfbaB	A 822 bp fusion cloned in pKMS1 for a 349 bp deletion in *fbaB*, Km^r^	This work
pCfbaB	pHM1 expressing *fbaB* under its own promoter, Sp^r^	This work
pfbaBaGUS	pHM1 expressing *gusA* under the *fbaB* promoter, Sp^r^	This work
pfbaBbGUS	pHM1 expressing *gusA* under the site (b)-mutated promoter of *fbaB*, Sp^r^	This work
pfbaBcGUS	pHM1 expressing *gusA* under the site (c)-mutated promoter of *fbaB*, Sp^r^	This work
pfbaBdGUS	pHM1 expressing *gusA* under the site (d)-mutated promoter of *fbaB*, Sp^r^	This work

a Ap^r^ = ampicillin resistance, Km^r^ = kanamycin resistance, Rif^r^ = rifampicin resistance, Sp^r^ = spectinomycin.

### DNA manipulation

DNA manipulation was performed following the standard procedures described by Sambrook [51]. The transconjugation between the *X. oryzae* pv. *oryzicola* and plasmids was performed as described by Turner [52]. Restriction enzymes and DNA ligases were performed in accordance with the manufacturer's instructions (Takara, Dalian, China). The PCR primers (Table S1) for gene targets in this report were purchased from Jinsite Biotechnology (http://www.croasia.net/company/jinsite_biotechnology_co.html). The genes cloned or amplified in this study were refered to the *hrp* clusters of *X. oryzae* pv. *oryzicola* RS105 strain (AF272885, AY875714) and the genome sequence of *X. oryzae* pv. *oryzicola* BLS256 strain (http://cmr.jcvi.org/cgi-bin/CMR/GenomePage.cgi?org=Xoc).

### Rice suspension cell cultures

*Oryza sativa* ssp. *indica* cv. Shanyou63, susceptible to *X. oryzae* pv. *oryzicola* RS105 strain, was used for callus induction. Seeds were dehulled and sterilized in 70% ethanol for 10 min and then in 50% commercial bleach with a few drops of Tween-20 for 30 min and then in 1% HgCl_2_ for 15 min. The sterilized seeds were washed 5 times with sterile distilled water and placed on N_6_ medium (10) with 2, 4-D (5 mg/L) for induction of rice callus at 28°C in the dark. The actively growing calli were selected and transferred to liquid N6 medium containing with 5 mg/L 2, 4-D and 1 mg/L kinetin (KT). The cells were maintained in the dark on a 7 day subculture schedule at a dilution of 1∶5 (inoculum: fresh medium). Generally, large amounts of rice suspension cells can be obtained after 4–5 week subculture and then dispersed or single round rice cells could be observed under the microscope.

### Construction of a non-polar mutant in *fbaB* of *X. oryzae* pv. *oryzicola*

The non-polar mutant of *fabB* in *X. oryzae* pv. *oryzicola* RS105 strain was constructed by using homologous recombination as described by Jiang [53], using pKMS1 as a suicide vector. Two flanking fragments, left and right to *fabB* (Figure S1), were amplified using the genomic DNA of strain RS105 as the template and the primers fbaBI-F/fbaBI-R and fbaBII-F/fbaBII-R (Table S1), respectively, and then cloned into pMD18-T vectors (Takara, Dalian, China), respectively. After confirmed by sequencing, the two fragments were digested and cloned into the vector pKMS1 at *Bam*HI and *Pst*I sites, resulting in pKΔfbaB (Table 1). The plasmid pKΔfbaB was introduced into RS105 by electrotransformation, and then the electrotransformants were plated on NAN plates supplemented with kanamycin. The emerged colonies suggested that the first homologous crossover event occurred in the electron transformants in which the DNA of the deletion vector was integrated into either the left or the right border of *fbaB* in the recipient chromosome (Figure S1). The single colonies of the mutant produced by single homologous crossover event were then transferred to NBN broth to culture for 12 h at 28°C. Then the bacterial cell was plated on NAS plates. The single colonies emerged within 3–4 day were then picked up into NA and NA plus kanamycin plates, respectively. By contrast, these kanamycin sensitive colonies could be the mutants in which the second homologous crossover event occurred and then were confirmed by PCR amplification with the primer pair fbaBI-F/fbaBII-R (Table S1, Figure S1). Subsequently, Southern hybridization (DIG, Roche) was conducted to verify the deletion of the *fbaB* by using the left fragment as the probe (Figure S1). One of the confirmed mutants, RΔfbaB (Table 1), was used for further study.

### Complementation of the *fbaB* mutant RΔ*fbaB*

In order to complement the *fbaB* mutant R*ΔfbaB*, a 1302 bp DNA fragment containing the entire *fbaB* gene (from 297 bp upstream of the start codon to the stop codon) was amplified by PCR using the total DNA of *X. oryzae* pv. *oryzicola* RS105 as the template and the primer pair fbaB-F/fbaB-R (Table S1). After being confirmed by sequencing, the amplified DNA fragment was cloned into pHM1 vector at *Hin*dIII and *Kpn*I sites to create a recombinant plasmid pCfbaB (Table 1). Plasmid pCfbaB was then transferred into the R*ΔfbaB* strain by electroporation. The transconjugants carrying pCfbaB were screened on NA plates with rifampicin and spectinomycin. A confirmed representative was verified by colony-PCR amplification for further study and named CRΔ*fbaB* (Table 1).

### HR and pathogenicity assays

HR and pathogenicity assays were performed as described [19]. *Xanthomonas* bacteria were grown in NB liquid with appropriate antibiotics at 28°C with shaking at 200 rpm for 16 h. The bacterial inocula were washed twice and resuspended in sterile water to 1×10^8^ cfu/ml and used to infiltrate into tobacco leaves (Xanthi) for HR detection and into rice seedlings (cv. Shanyou63, susceptible to *X. oryzae* pv. *oryzicola* infection, two-week old) for water-soaking formation with needleless syringes, respectively, and to inoculate in adult rice plants (cv. Shanyou63, two-month old) by leaf-needling for lesion length measurement. All plants were grown and maintained in a greenhouse with 12-h day-night cycle illuminations with a fluorescent lamp and a constant temperature of 25°C with relative humidity at 75 to 80% [54]. Plant responses were scored at 24 h for HR, in 3 days for water-soaking symptoms, and in 14 days for lesion lengths after inoculation. Five leaves were inoculated for each independent experiment, and each treatment was repeated three times.

### Determination of bacterial growth ability *in planta* and in minimal medium supplemented with different carbohydrates

*Xanthomonas* bacterial suspensions at 1×10^8^ cfu/ml were infiltrated into the intercellular spaces of fully expanded leaves of rice (cv. Shanyou63, two-week old) with needleless syringes at three spots on each leaf. Three 0.8 cm diameter leaf discs were harvested with a cork borer from each infiltration area after infiltration. After being sterilized in 70% ethanol and 30% hypochlorite, the leaf discs were homogenized in 1 ml of distilled water. Diluted homogenates were plated on NA agar plates supplemented with appropriate antibiotics. The number of bacterial colonies on these plates was counted after incubation at 28°C until single colonies could be counted after 3 to 4 days. The number of bacterial CFU per square centimeter of leaf area was then estimated, and the standard deviation was calculated using colony counts from the three triplicate spots from each of the three samples per time point per inoculum. Experiments were repeated at least three times.

As to the detection of bacterial growth influenced by different carbohydrates, *Xanthomonas* bacteria were preincubated in 5 ml NB medium for 16–20 h at 28°C with shaking at 200 rpm until the OD_600_ value reached 0.6, and 2% of this culture was subcultured into 20 ml of the fresh NB for 16–18 h incubation. The bacterial cells were collected and washed twice, and resuspended to an optical density of 600 nm of 0.1 in 100 ml of the minimal medium NCM supplemented with different carbon source at 0.5%. For each time point, 200 ul of each culture was removed and determined by measuring OD_600_ against the medium blank. Data presented were from a representative experiment; the experiment was repeated independently three times.

### Site-directed mutagenesis in the PIP-box motifs of *fbaB* by PCR amplification

There is a PIP-box sequence, TTCGT-N_9_-TTCGT, which is not typical to TTCGC-N_15_-TTCGC, following 30 base pair interval space before a -10 box-like motif CAGCAT in the *fbaB* promoter region. Base-substitution in the PIP-box sequence of the *fbaB* promoter region was performed via a PCR amplification strategy. Briefly, three substituted sequences, TTCGC-N_9_-TTCGT (fbaBb), TTCGT-N_9_-TTCGC (fbaBc) and TGATA-N_9_-TTCGT (fbaBd) within the *fbaB* promoter region were generated by using three primer pairs, fbaBa-F/fbaBb-R, fbaBa-F/fbaBc-R and fbaBa-F/fbaBd-R (Table S1), respectively, for PCR amplification with the genomic DNA of *X. oryzae* pv. *oryzicola* RS105 as the template. Then, these PCR products were cloned into pMD_18_-T vector and confirmed by sequencing for further study.

### Construction of the *fbaB* reporter plasmids

To investigate whether the expression of *fbaB* is or not regulated by HrpG and HrpX, four *fbaB* reporter plasmids, pFbaBaGUS, pFbaBbGUS, pFbaBcGUS and pFbaBdGUS, were constructed by cloning the PIP-box promoter region and three mutated PIP-box promoters of the *fbaB* gene which were fused with the promoterless β-glucuronidase (*gusA*) gene into the broad-host-range cloning vector pHM1 (Table 1) at MCS (multiple cloning site). A 366 bp region upstream of the *fbaB* was amplified by PCR using the total DNA of the wild-type RS105 strain as the template and the primer pair FbaBPF/FbaBPR (Table S1). The amplified fragment of the wild-type promoter of *fbaB*, confirmed by sequencing, was fused with the promoterless *gusA* in the vector pHM1 at *Hind*III and *Eco*RI sites to create the recombinant plasmid pFbaBaGUS (Table 1). In contrast, the wild-type promoter was replaced by three site-directed substitutes in the PIP-box of the *fbaB* promoter, as mentioned above, and fused with the *gusA* gene in the vector pHM1 at *Hind*III and *Eco*RI sites, generating the recombinant plasmids pFbaBbGUS, pFabBcGUS and pFbaBdGUS (Table 1). The plasmids obtained were further confirmed by restriction analysis and sequencing.

### Measurement of EPS production

EPS production was measured as previously described by Tang [55]. In brief, *Xanthomonas* bacteria were grown in 100 ml of NY medium supplemented with 2% (w/v) various sugars at 28°C with constant shaking at 200 rpm for 3 days. EPS was precipitated from the culture supernatant with ethanol, and dried to constant weight at 55°C, and weighed. Every experiment was repeated at least three times.

### Semi-quantitative RT-PCR and Real-time quantitative RT-PCR

The expression of tested genes, including the reporter *gusA*, was assayed by semi-quantitative RT-PCR or real-time quantitative RT-PCR with corresponding primer pairs (Table S1). *Xanthomonas* bacteria were preincubated in 20 ml NB medium for 16–20 h, until the OD_600_ value reached 0.6, and 2% of this culture was subcultured into 20 ml of the fresh NB for 16–18 h incubation. The bacterial cells were collected and washed twice, and resuspended to an optical density of 600 nm of 2.0 by sterilized water. Then, 40 ul of bacterial suspension was inoculated into 1.5 ml of NB, NY medium or rice suspension cells incubating for 16 h at 25°C. As a template, total RNAs were extracted using the Trizol reagent (Takara, Dalian, China) according to the manufacturer's protocol. cDNA synthesis was conducted with AMV random primers (order no. D3801) provided by the manufacturer (Takara, Dalian, China). Before synthesis of the first-strand cDNA, total RNAs were digested with RNase-free DNase I (TaKaRa, Dalian, China) to remove potential traces of genomic DNAs. Semi-quantitative RT-PCR was performed on the ordinary PCR apparatus and the PCR program was as follows: step 1, 95°C for 3 min; step 2, 95°C for 20 s; step 3, 55°C for 30 s; step 4, 72°C for 40 s; 35 cycles from steps 2 to 4; and step 5, 72°C for 10 min. Real-time quantitative RT-PCR was performed on the Applied Biosystems 7500 real-time PCR System using SYBR *Premix Ex Taq*™ (Takara, Dalian, China), and the PCR thermal cycle condition was as following: denature at 95°C for 30 s and 41 cycles for 95°C, 5 s; 60°C, 34 s. The expression level of the *16S rRNA* gene was used as an internal standard. The comparative-threshold method was used to calculate the relative mRNA level with respect to the corresponding transcript in cells cultured in NB or NY medium or rice suspension cells. All RT-PCRs were performed in triplicate.

## Results

### *fbaB* is required for full virulence and growth of *X. oryzae* pv. *oryzicola in planta*

The discovery of the *fbaB* gene as a virulence factor came from work aimed at the identification of genes involved in virulence alteration of *X. oryzae* pv. *oryzicola* RS105 strain in rice. The approach was to screen a Tn5-tagged mutant library of RS105 [20] for mutants that could impair virulence of the bacterium in rice. One mutant Mxoc0504 (Table 1), where the Tn5 transposon was inserted in an open-reading frame (ORF) of *Xoryp_17640* at the 185 bp site (Figure S1), reduced virulence of *X. oryzae* pv. *oryzicola* RS105 in rice (data not shown). The genome location and genetic organization of the Tn5-insertion in *Xoryp_17640* of *X. oryzae* pv. *oryzicola* RS105 suggests the presence of a transcriptionally active gene *Xoryp_17640* (Figure S1). Fructose-bisphophate aldolase is an enzyme encoded by just one gene, which is highly conserved in *Xanthomonas* species (data not shown). It performs the reversible action of converting fructose-1,6-bisphosphate to dihydroxyacetone phosphate and glyceraldehyde 3-phosphate, which are involved in functional glycolytic and gluconeogenic pathways [1]. Thus, *Xoryp_17640* is hereafter named as *fbaB*.

To faciliate the functional study of *fbaB*, a nonpolar *fbaB* mutant, named RΔ*fbaB* (Table 1), was constructed by homologous suicide plasmid integration (Figure S1) (see Materials and Methods for detail). A complemented strain named CRΔ*fbaB* was also constructed by introducing the recombinant plasmid pCfbaB, which carries the entire ORF *fbaB* with a 297 bp promoter region upstream of the start codon (Table 1), into the mutant RΔ*fbaB*.

**Figure 1:**
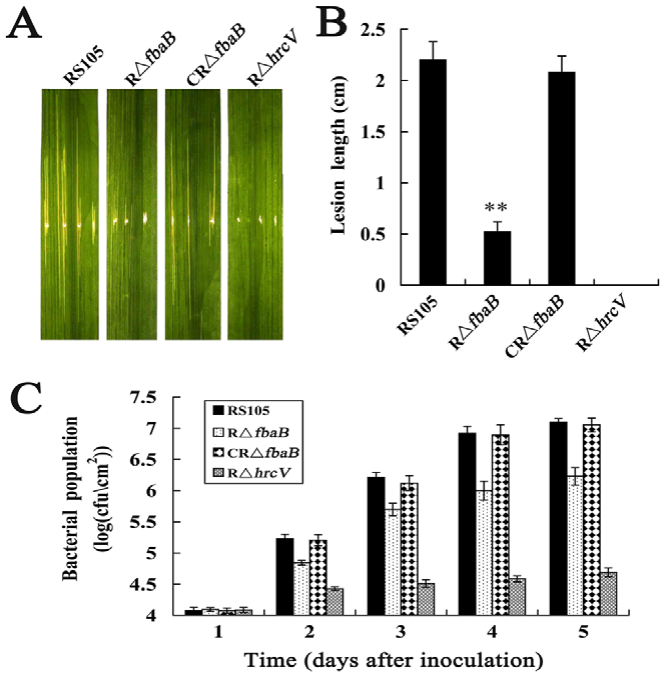
*fbaB* is required for full virulence and growth of *X. oryzae* pv. *oryzicola in planta*. (A) Symptoms caused by different *X. oryzae* pv. *oryzicola* strains suspended in water to OD_600_ = 0.3 (approximately 1×10^8^ cfu/ml) on inoculated leaves of the host rice cv. Shanyou63 (susceptible cultivar) (2-month-old) by leaf-needling inoculation. Photographs were taken 14 days post-inoculation. RS105, the wild-type strain; RΔ*fbaB*, the *fbaB* deletion mutant; CRΔ*fbaB*, the complemented strain of RΔ*fbaB* with the *fbaB* gene; RΔ*hrcV*, a type III-deficient strain as a negative control. (B) Lesion lengths of rice bacterial leaf streak caused by different *X. oryzae* pv. *oryzicola* strains in rice. Values are the means ± standard deviations (SD) from three repeats, each with five leaves. The different symbol in each horizontal data column results from a paired, two-tailed Student *t* test relative to the wild-type. **, P = 0.01. (C) Bactrial growth capacity in inoculated leaves. Bacteria were recovered from the inoculated leaves every 24 hours in a period of 4 days post inoculation, and homogenized in sterile water. The homogenates were diluted and plated on NA plates with appropriate antibiotics. Bacterial CFU were counted after incubation at 28°C for 3 days. Data are the mean ± SD from three repeats.

The virulence of the *fbaB* mutant RΔ*fbaB*, the complemented strain CRΔ*fbaB* and the wild-type RS105 was tested on the hybrid rice cultivar Shangyou63 by the leaf-needling inoculation method [4]. Although the mutant strain RΔ*fbaB* still caused obvious bacterial leaf streak (BLS) symptoms, the symptoms were significantly less severe than that caused by the wild-type RS105 (Figure 1A). The mean lesion length caused by RΔ*fbaB* was significantly reduced (P = 0.01, *t* test) by approximately 1.5 cm compared to the wild-type RS105, while the T3SS mutant RΔ*hrcV*, used as a negative control, did not cause any BLS symptoms in rice (Figure 1A). The BLS lesion lengths caused by the complemented strain CRΔ*fbaB* were however obiviously the same as those caused by the wild-type RS105 (Figure 1B). These results demonstrated that *fbaB* is required for full virulence of *X. oryzae* pv. *oryzicola* in rice.

In order to determine whether *fbaB* results in a decrease in the proliferation of *X. oryzae* pv. *oryzicola* in the host rice, we investigated the growth capacity of the *fbaB* mutant R*ΔfbaB*, the complemented strain CR*ΔfbaB* and the wild-type strain RS105 *in planta*. During the observation days, the bacterial number of the R*ΔfbaB* mutant recovered from the infected rice leaves was significantly lower than that of the wild-type RS105 at each of the test points. The growth capacity of the R*ΔfbaB* strain *in planta* was completely restored to the wild-type level by *fbaB in trans* (Figure 1C), whereas the T3SS mutant RΔ*hrcV* did not grow more in inoculated rice tissues. These results indicated that the *fbaB* is required for growth of *X. oryzae* pv. *oryzicola in planta*.

### *fbaB* is important in acquisition of fructose, pyruvate and malate for *X. oryzae* pv. *oryzicola* growth

**Figure 2:**
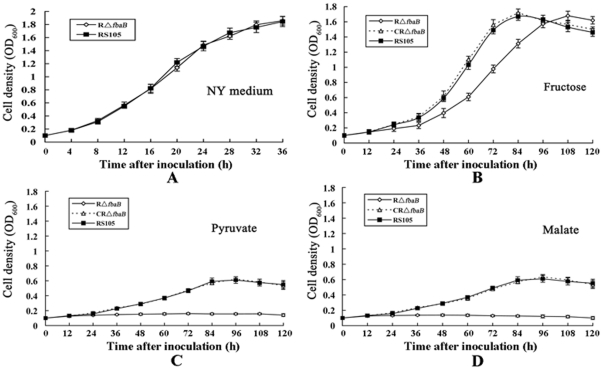
Growth curves of *X. oryzae* pv. *oryzicola* in sole carbon media. RS105, the wild-type strain; RΔ*fbaB*, the *fbaB* deletion mutant; CRΔ*fbaB*, the complemented strain of RΔ*fbaB* with the *fbaB* gene. The initial concentration of the tested strains was adjusted to OD_600_ of 0.1 with NCM supplemented with fructose, pyruvate or malate as the sole carbon source. Aliquots were taken in triplicate at intervals of 120 h after incubation at 28°C, and bacterial growth was determined by measuring OD_600_ against the medium blank. Values given are the means ± SD of triplicate measurements from a representative experiment; similar results were obtained in two other independent experiments.

FbaB reversibly converts fructose-1,6-bisphosphate to dihydroxyacetone phosphate and glyceraldehyde 3-phosphate. This prompted us to investigate whether *fbaB* affects *X. oryzae* pv. *oryzicola* growth in a non-sugar NY medium (see Materials and Methods for detail). The result showed that the *fbaB* mutant R*ΔfbaB* grew identically as the wild-type RS105 (Figure 2A), indicating that the R*ΔfbaB* mutant was not auxotrophic. To further examine the effect of the *fbaB* gene on the ability of *X. oryzae* pv. *oryzicola* to utilize various carbon sources, the growth of the *fbaB* mutant R*ΔfbaB*, the complemented strain CR*ΔfbaB* and the wild-type RS105 were tested by using the liquid NCM (non-carbohydrate minimal medium) supplemented with glucose, sucrose, fructose, mannose, galactose, pyruvate and malate, respectively, as the sole carbon source. The growth of the R*ΔfbaB* strain grew in a similar fashion to that of the wild-type RS105 when supplemented with glucose, sucrose, mannose or galactose (data not shown). However, the growth of R*ΔfbaB* was significantly slower than that of the wild-type strain RS105 in liquid NCM supplemented with fructose as the sole carbon source, while the complemented strain CR*ΔfbaB* with the *fbaB* gene restored the growth to the wild-type level (Figure 2B), suggesting that the mutation in *fbaB* diminishes the capability of *X. oryzae* pv. *oryzicola* to utilize fructose.

Since pyruvate is the final product in glycolysis, and the initial carbohydrate for gluconeogenesis [1], the mutation in *fbaB* may presumably lead no gluconeogenesis. To seek this, we then investigated whether the mutagenesis in *fbaB* causes *X. oryzae* pv. *oryzicola* unable to utilize pyruvate or not. Indeed, the *fbaB* mutant R*ΔfbaB* was unable to grow in NCM medium supplemented with pyruvate as the sole carbon source, whereas the complemented strain CR*ΔfbaB* harboring the *fbaB* gene restored the ability to acquire pyruvate to the wild-type level (Figure 2C).

Pyruvate may be catalyzed by pyruvate carboxylase into oxaloacetate, or by the pyruvate dehydrogenase complex into acetyl-CoA which essentially flows into the TCA cycle. Malate is reversibly converted by malic enzyme into pyruvate for gluconeogenesis [1]. This promopted us to investigate whether or not the mutation in *fbaB* impairs the ability of *X. oryzae* pv. *oryzicola* to utilize malate for growth. The results showed that the *fbaB* mutant R*ΔfbaB* was unable to grow in NCM supplemented with malate as the sole carbon source, while the complemented strain CR*ΔfbaB* with *fbaB* was recovered to the wild-type level to use malate for growth (Figure 2D), implying that the conversion of malate into pyruvate can not flow through gluconeogenesis because of the mutation in *fbaB*.

Briefly, the above data indicate that *fbaB* of *X. oryzae* pv. *oryzicola* has limited influence on fructose utilization due to the presence of ED and PPP pathways when the downstream glycolysis is blocked, but plays important roles in gluconeogenesis when malate from TCA cycle is converted into pyruyate.

### *fbaB* influences EPS production of *X. oryzae* pv. *oryzicola*

It has been demonstrated that EPS as a virulence factor plays an important role during bacterial infection [56], [57] and ED is necessary for xanthan biosynthesis and PPP does not influence xanthan biosynthesis in *X. oryzae* pv. *oryzae* [6]. In order to determine whether the mutation in *fbaB* has any effect on EPS production of *X. oryzae* pv. *oryzicola*, the EPS yields of the *fbaB* mutant R*ΔfbaB*, the complemented strain CR*ΔfbaB* and the wild-type RS105 were quantitatively measured after the strains grew in NY liquid medium supplemented with 2% of fructose, pyruvate and malate, respectively, for 3 days. Meanwhile, NY medium and NY medium added with 2% of glucose were used as the control. After the EPS of the tested strains was extracted from the cultures (see Materials and Methods for detail), we found that there were no significant (P = 0.01, *t* test) difference in EPS production among R*ΔfbaB*, CR*ΔfbaB* and RS105 when they grew in NY medium alone and NY medium containing 2% of glucose, respectively (Table 2). However, the EPS yield of R*ΔfbaB* was significantly 30% less than that of the wild-type RS105 in NY plus 2% fructose medium, 80% less in NY plus pyruyate or malate (Table 2). By contrast, the *fbaB* gene restored EPS production of the mutant R*ΔfbaB* to the wild-type level either in NY plus 2% fructose, or NY plus 2% pyruvate, or NY plus 2% malate (Table 2). The above results suggest that the mutation in *fbaB* leaves *X. oryzae* pv. *oryzicola* unable to sufficiently use fructose, and to completely uitilize pyruvate and malate. This provides evidence that the *fbaB* mutation on gluconeogenesis affects the ED pathway, resulting in less EPS production.

**Table 2:** EPS products in *X. oryzae* pv. *oryzicola* strains.

Strains^a^	EPS yield (g/100 ml)^b^				
	NY	NY plus 2% glucose	NY plus 2% fructose	NY plus 2% pyruvate	NY plus 2% malate
R*ΔfbaB*	0.05±0.009^A^	0.88±0.082^A^	0.52±0.012^A^	0.12±0.012^A^	0.10±0.009^A^
CR*ΔfbaB*	0.06±0.006^A^	0.94±0.032^A^	0.81±0.028^B^	0.49±0.023^B^	0.54±0.036^B^
RS105/pHM1	0.05±0.007^A^	0.91±0.026^A^	0.75±0.034^B^	0.53±0.019^B^	0.50±0.025^B^

a Strains were cultured in NY medium alone and supplemented with 2% various carbon sources.

b Data presented are the means ± standard deviations of triplicate measurements from a representative experiment, and similar results were obtained in two other independent experiments. Different letters in each data column indicate significant differences (P = 0.01; *t* test).

### *fbaB* is negatively regulated by HrpX and HrpG

**Figure 3:**
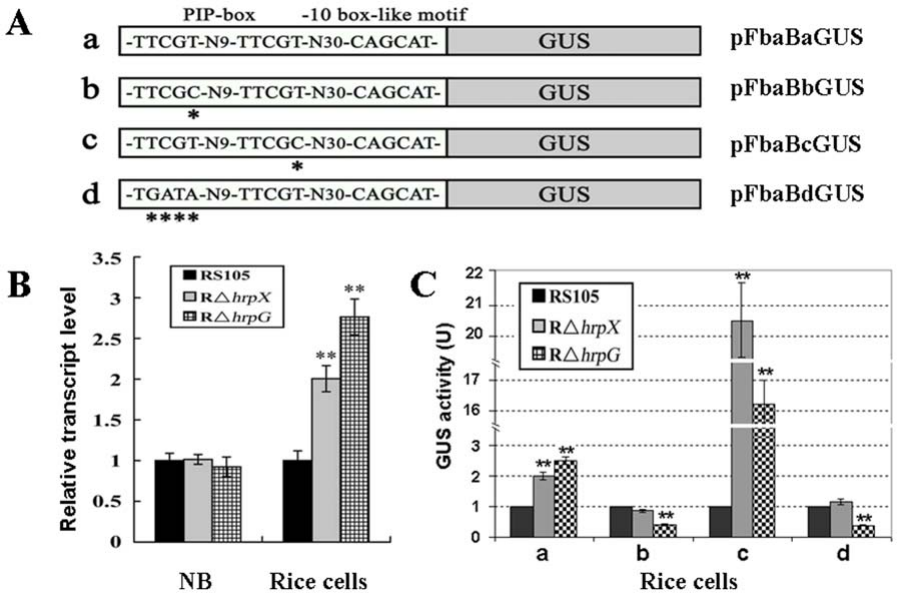
The expression of *fabB* in *X. oryzae* pv. *oryzicola* is negatively by *hrpX* and *hrpG* in rice suspension cells. (A) Schematic map of the promoter region containing PIP-box and -10 box-like motif of *fabB* fused with a promoterless *gusA* gene. * stands for base substitutions. The constructs are listed on the right. (B) Expression analysis of *fbaB* by real-time quantitative RT-PCR. RNAs were isolated from cultures of the wild-type RS105, the *hrpG* deletion mutant RΔ*hrpG* and the *hrpX* deletion mutant RΔ*hrpX* strains which were grown in NB medium and rice suspension cells for 16 h, respectively. The relative mRNAs level of *fbaB* was calculated with respect to the level of the corresponding transcript in the wild-type RS105. (C) Effects of the mutated PIP-box on *gusA* transcript. The *gusA* transcript level by the wild-type PIP-box promoter (a) and three base-substituted PIP-box promoter (b, c, d) in the wild-type RS105, the *hrpX* mutant RΔ*hrpX* and the *hrpG* mutant RΔ*hrpX* were investigated, respectively. All the reporter strains above were cultured in rice suspension cells for 16 h and *gusA* transcript levels were then determined by real-time PCR. The transcript of *gusA* in the wild-type was taken as one unit. Data are the mean ± SD of triplicate measurements from a representative experiment; and similar results were obtained in two other independent experiments. The asterisks in each horizontal data column indicate significant differences at P = 0.01 by *t* test.

Previous reports have demonstrated that the PIP-box of HrpX regulons serves as a *cis*-regulated element in a HrpX-dependent manner [37], [38], [44]. Analysis of the promoter region of *fbaB* of *X. oryzae* pv. *oryzicola* BLS256 (Figure S1) by searching the existence of similar PIP-box sequence and by using a promoter-prediction software (http://www.fruitfly.org/seq_tools/promoter.html) revealed an imperfect PIP-box (TTCGT-N_9_-TTCGT) interval by 30 bp sequence with a -10 box-like motif (CAGCAT) upstream of the *fbaB* start codon (Figure 3A), suggesting that the expression of *fbaB* may be regulated by HrpX and HrpG, the latter controls the expression of *hrpX* [34], [35]. To investigate this, a real-time quantitative RT-PCR was employed to assay the action of the *fbaB* transcript with *hrpX* and *hrpG*. The *fbaB* relative transcript level displayed a significant increase (P = 0.01, *t* test) in the *hrpX* mutant R*ΔhrpX* and the *hrpG* mutant R*ΔhrpG* than that of the wild-type RS105 when the strains grew in rice suspension cells for 16 h. The expression of *fbaB* in R*ΔhrpG* was higher than that in R*ΔhrpX*, whereas there were no obvious difference of the *fbaB* expression among these three tested strain when they grew in NB medium (Figure 3B). These results demonstrate that the expression of *fbaB* is inducible *in planta* and repressed by HrpX and HrpG when the pathogen infects the host rice rather than in necrotrophic growth. The negative regulation of *fbaB* with HrpG and HrpX and the higher expression of *fbaB* in the *hrpG* mutant than in the *hrpX* mutant imply that other unkown factor(s) may involve in the regution of *fbaB*.

It has been demonstrated that HrpX regulates the expression of HrpX regulon genes by binding the PIP-box motif in promoter regions [36], [38], [39]; therefore, substitution of the fifth base in the motif TTCGC, or the complete mutation in the motif sequence itself, significantly alters promoter activity [26], [37], [38]. Fifth base substitution, TTCG**B** (B = T, C, or G) may increase or decrease the transcript level of a HrpX-regulated gene by up to 50% [38], [39]. In order to determine whether or not the two motifs of the imperfect PIP-box (TTCGT-N_9_-TTCGT) of *fbaB* are affected by HrpG and HrpX, the fifth base of the first or second TTCGT motif was substituted with a C to create TTCG**C** by site-direct substitutions, which generated pFbaBbGUS and pFbaBcGUS (Figure 3A), respectively. In addition, the first TTCGT motif was completely changed to a TGATA motif to produce pFbaBdGUS (Figure 3A) by using site-mutagenesis primers (Table S1). The GUS reporter strains (Table 1) were incubated in rice suspension cells for 16 h. The *gusA* transcript level was measured by real-time PCR (Figure 3C). Compared to pFbaBaGUS, pFbaBbGUS andpFbaBdGUS (Figure 3A) reduced the *gusA* transcript level in R*ΔhrpX* (Figure 3C). This was similar to the *gusA* transcript level of pFbaBaGUS in the wild-type when compared to the *hrpX* mutant RΔ*hrpX* (Figure 3C). However, the *gusA* transcript of pFbaBbGUS or pFbaBdGUS in the *hrpG* mutant R*ΔhrpG* was significantly lower than that in the *hrpX* mutant or in the wild-type (Figure 3C). Intriguingly, the base substitution of the fifth residue in the right motif of the PIP-box (Figure 3A) significantly increased the *gusA* transcript of pFbaBcGUS in either R*ΔhrpX* or R*ΔhrpG*, compared with the wild-type (Figure 3C). The *gusA* expression of pFbaBcGUS in R*ΔhrpX* was significantly higher (approximately 20-fold higher than that in the wild-type) than that in R*ΔhrpG* (Figure 3C). The GUS activity assay also demonstrated the same results above (data not shown). The above evidence suggests that expression of *fbaB* is negatively regulated by HrpG and HrpX via the PIP-box promoter where a yet unknown factor might bind for the involved regulation.

### Various carbohydrates have different effects on expression of *hrpG*, *hrpX* and *fbaB* in *X. oryzae* pv. *oryzicola*

**Figure 4:**
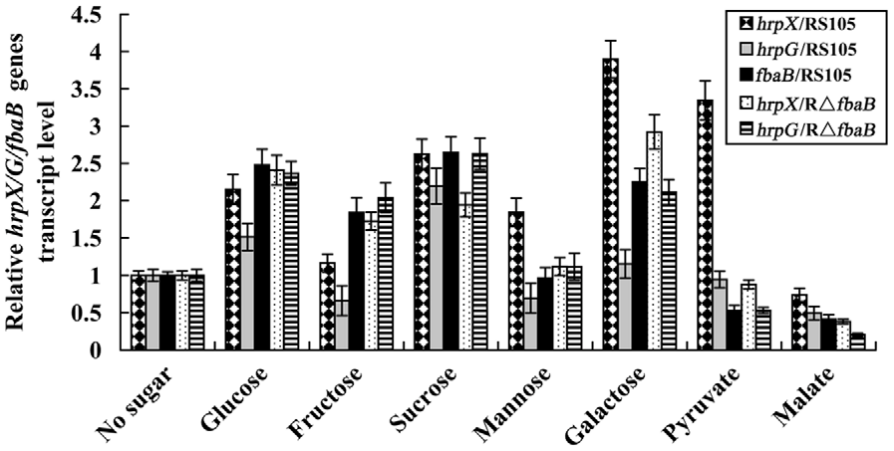
Effects of different carbohydrates on the expression of *hrpX*, *hrpG* and *fbaB* in *X. oryzae* pv. *oryzicola*. RNAs were isolated from cultures of the wild-type RS105 strain and the *fbaB* deletion mutant RΔ*fbaB* grown in NY medium alone and NY supplemented with 0.5% of various carbohydrates for 16 h. The relative mRNAs levels of *hrpX*, *hrpG* and *fbaB* genes were calculated by real-time quantitative RT-PCR with respect to the level of the corresponding transcript in the wild-type RS105 cultured in NY medium alone. Data presented are the means ± SD of triplicate measurements from a representative experiment; similar results were obtained in two other independent experiments.

Environmental signals like carbon sources presenting in plants may serve as inducers or inhibitors of virulence-associated gene expression in plant bacteria [17], [21]. We sought to investigate transcript production of *hrpG*, *hrpX* and *fbaB* when *X. oryzae* pv. *oryzicola* is fed with different carbohydrates. The expression level of *hrpG*, *hrpX* and *fbaB* was measured by real-time PCR after the wild-type RS105 strain grew for 16 h in NY medium complemented with 0.5% of sucrose, galactose, glucose, mannose, fructose, pyruvate and malate, respectively. Using NY medium as the control, we found that, besides malate, the other six carbon sources enhanced the expression of *hrpX*. Fructose, mannose, galactose, pyruvate and malate had little effect on the expression of *hrpG*, while sucrose and glucose increased the transcript level of *hrpG* (Figure 4). The transcript level of *fbaB* was increased by sucrose, galactose, glucose and fructose rather than pyruvate and malate (Figure 4). Noticeably, fructose, mannose and malate repressed the expression of *hrpG*, pyruvate and malate inhibited the expression of *fbaB*, and malate suppressed the transcript of *hrpX* (Figure 4). The results above demonstrate that expression of certain genes involving the carbon metabolic pathways may be regulated by the two key *hrp* regulatory genes *hrpG* and *hrpX*, and in turn the expression of *hrpG* and *hrpX* may also be enhanced or repressed by the carbon sources or intermediates in the metabolic pathways.

The negative regulation of *fbaB* with HrpG and HrpX prompted us to determine whether it is influenced by the carbohydrates tested in the previous section when *fbaB* is mutated. Using the same real-time PCR assay, we found that the relative mRNA level of *hrpX* was significantly reduced in the *fbaB* mutant than that in the wild-type RS105 strain when sucrose, mannose, galactose, pyruvate and malate were complemented in NY medium as the sole carbon sources, but increased when fructose was used. The expression of *hrpG* was significantly enhanced by glucose, fructose, sucrose, mannose and galactose, respectively, and repressed by pyruvate and malate (Figure 4). These results demonstrated that the mutation in *fbaB* may alter the expression of *hrpG* or *hrpX* when the sugars are used as the sole carbon source and the relationship of HrpX with HrpG may be influenced through unkown factor(s) affected by different carbonhydrates.

### *fbaB* positively affects the expression of *hrpG* and *hrpX*, but negatively influences the transcripts of *hrcC*, *hrp*E and *hpa3*

**Figure 5:**
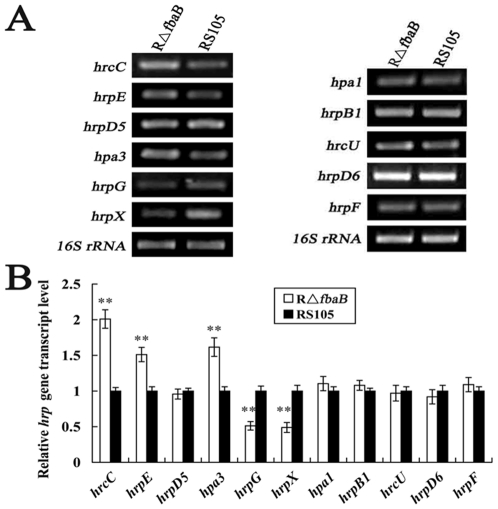
Influence of the mutation in *fbaB* on the expression of *hrp-hrc-hpa* genes of *X. oryzae* pv. *oryzicola*. (A) Semi-quantitative RT-PCR analysis. RNAs were isolated from cultures of the wild-type RS105 strain and the *fbaB* mutant RΔ*fbaB* grown in rice suspension cells for 16 h. The *16S rRNA* gene of the pathogen is used as the standard internal control. The tested *hrp-hrc-hpa* genes were selected based on the reports [19], [20], [53] with the primer pairs (Table S1) and the sequence of the *hrp* clusters (AF272885, AY875714) was used as the reference. (B) Real-time quantitative RT-PCR analysis. The relative mRNA level of the tested *hrp-hrc-hpa* genes in the *fbaB* mutant RΔ*fbaB* was calculated with respect to the level of the corresponding transcripts in the wild-type RS105 cultured in rice suspension cells for 16 h. Values given are the means ± SD of triplicate measurements from a representative experiment. The asterisks in each horizontal data column indicate significant differences. **, P = 0.01, *t* test. Experiment was repeated twice and yielded similar results.

The data above demonstrated that expression of the key *hrp* regulatory genes, *hrpG* and *hrpX*, are induced or repressed when *X. oryzae* pv. *oryzicola* evidently uses sugars from plants as nutrient sources, implying that the expression of the *hrp*-*hrc*-*hpa* genes, which are regulated by HrpG and HrpX as reported [20], may be altered when *fbaB* is mutated. To investigate this, we employed a semi-quantitative (Figure 5A) and real-time RT-PCR (Figure 5B) with the specified primer pairs (Table S1) to evaluate the transcript production of representative *hrp-hrc-hpa* genes in the *fbaB* mutant R*ΔfbaB* and the wild-type RS105 strain after incubation in rice suspension cells for 16 h, while the nutrient-rich medium NB was used as a synchronous control (data not shown). The results showed that: i) the expression of *hrpG* and *hrpX* in the *fbaB* mutant RΔ*fbaB* was significantly (P = 0.01, *t* test) lower than that in the wild-type, implying that the dysfunction in glycolysis and gluconeogenesis by the mutation in *fbaB* represses the transcript of *hrpG* and *hrpX*; ii) the mRNA level of the tested genes, *hrpD5*, *hrpD6*, *hpa1*, *hrpB1*, *hrcU* and *hrpF*, in R*ΔfbaB* was similar to that in the wild-type; iii) the transcriptional level of *hrcC*, *hrpE* and *hpa3* (which was previously reported that their expression was not completely controlled by HrpG and HrpX in *X. oryzae* [24], [31]) in R*ΔfbaB* was significantly higher than that in the wild-type (Figure 5), indicating that certain intermediates in glycolysis and gluconeogenesis derived from the aldol reaction [1] may influence the expression of *hrcC*, *hrpE* and *hpa3*. The above data suggest that the mutation in *fbaB* may alter the ability of *X. oryzae* pv. *oryzicola* to acquire carbon from its living niche which in turn represses the expression of *hrpG* and *hrpX*.

### *hrcC*, *hrpE* and *hpa3* are required in utilization of pyruvate and malate for *X. oryzae* pv. *oryzicola*

**Figure 6:**
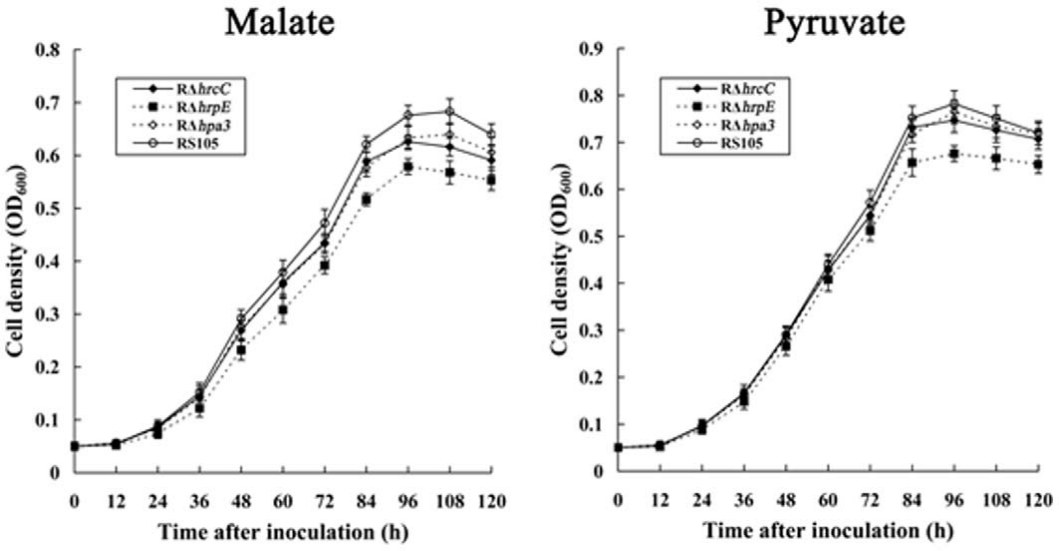
The mutation in *hrcC*, *hrpE* and *hpa3* reduced the ability of *X. oryzae* pv. *oryzicola* to acquire pyruvate and malate. RS105, the wild-type strain; RΔ*hrcC*, the *hrcC* deletion mutant; RΔ*hrpE*, the *hrpE* deletion mutant; RΔ*hpa3*, the *hpa3* deletion mutant. The initial concentration of the tested strains was adjusted to OD_600_ of 0.05 with NCM supplemented with pyruvate or malate as the sole carbon source. Aliquots were taken in triplicate at intervals of 120 h after incubation at 28°C, and bacterial growth was determined by measuring OD_600_ against the medium blank. Values given are the means ± SD of triplicate measurements from a representative result of other two similar independent experiments.

To verify our hypothesis that the *hrcC*, *hrpE* and *hpa3* genes are involved in acquisition of pyruvate and malate, we tested the growth of the *hrcC*, *hrpE* and *hpa3* mutants, RΔ*hrcC*, RΔ*hrpE*, and RΔ*hpa3* (Table 1), respectively, in NCM medium supplemented with sucrose, mannose, galactose, glucose, fructose, pyruvate and malate as the sole carbon sources, while the wild-type strain RS105 was used as the control. Indeed, the mutation in *hrcC*, *hrpE* or *hpa3* reduced the growth of the pathogen when pyruvate and malate were used as the sole carbon source (Figure 6). By contrast, the growth of the *hrpE* mutant was affected much more than that of the *hrcC* or the *hpa3* mutants by pyruvate and malate, respectively (Figure 6). These data suggests possible reasons why the expression of *hrcC*, *hrpE* and *hpa3* was higher than other *hrp-hrc-hpa* genes when *fbaB* was mutated (Figure 5).

## Discussion

**Figure 7:**
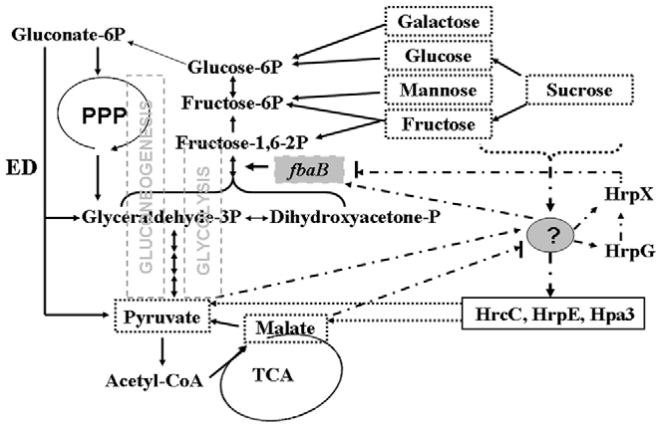
Working model of FbaB coordinating with *hrp* genes of *X. oryzae* pv. *oryzicola* in carbon metabolic pathoways. The lined arrows from the carbohydrates in dashed-line boxes or the double lined arrows from the intermediates indicate carbon flows in glycolysis, gluconeogenesis, pentose phosphate pathway (PPP), entner–doudoroff (ED) and tricarboxylic acid (TCA) cycle pathways, respectively. The grey box displays *fbaB* encodes a fructose-bisphophate aldolase that converts vertically fructose-1,6-bisphosphate to dihydroxyacetone phosphate and glyceraldehyde 3-phosphate. The mutation in *fbaB* has no influence on ED and PPP pathways, but impairs glycolysis of the pathogen to use fructose and block gluconeogenesis to use pyruvate and malate. The expression of an unknown regulator in a cycled question mark may be enhanced by galactose, glucose, mannose, sucrose, fructose and pyruvate (as shown by dash-lined arrow) and repressed by malate (a dash-lined arrow with a stop bar). The unkown factor may differentially regulate the expression of *hrpG* or/and *hrpX* which down-regulate the expression of *fbaB* (a dash-lined arrow with a stop bar). The unknown regulator may also control the transcripts of *hrcC*, *hrpE* and *hpa3* (other than other *hrp-hrc-hpa* genes) which are not completely regulated by HrpG and HrpX [20]. Being the components of the T3SS apparatus, HrcC, HrpE and Hpa3 may faciliate *X. oryzae* pv. *oryzicola* to utilize the intermediates, like pyruvate and malate, of the TCA cycle from plants.

In this study, we identified in *X. oryzae* pv. *oryzicola* RS105 strain a novel and unique virulence gene, *fbaB*, which encodes an fructose-bisphophate aldolase (FbaB), highly conserved in other Xanthomonads, and converts the intermediate fructose-1,6-bisphosphate to reversible dihydroxyacetone phosphate and glyceraldehyde 3-phosphate, which is essential for glycolysis and gluconeogenesis (Figure 7), and now has been shown to play a role in virulence. Genetic evidence presented here demonstrates that *fbaB* is required for *X. oryzae* pv.*oryzicola* to utilize fructose, pyruvate and malate so that the pathogen produces EPS and expands full virulence and growth *in planta* for adaptation. The mutation in *fbaB* does not make the pathogen auxotrophic and lethal. Interestingly, the expression of *fbaB* is negatively regulated by the HrpG and HrpX cascade via the imperfect PIP-box of *fbaB* possibly with an unknown regulator. The latter may presumably regulate the expression of *hrcC*, *hrpE*, and *hpa3* and be influenced by the accumulation of pyruvate for the initiation of gluconeogenesis and malate from the TCA cycle (Figure 7). Intriguingly, the PIP-box spaced, by 30 base pairs with a -10 box-like motif is also highly conserved within the genome sequences of *X. oryzae* pv. *oryzae* PXO99^A^ [9], KACC10331 [8], *X. campestris* pv. *vesicatoria* 85-10 strain [12], *X. campestris* pv. *campestris* 8004 [10], and *X. axonopodis* pv. *citri* 306 [11] (data not shown). This implies that the expression of *fbaB* homologues in other *Xanthomonas* species may be regulated by the same manner. However, whether *fbaB* of other *Xanthomonas* species plays a similar role as above in host-pathogen interactions needs to be further investigated.

*X. oryzae* pv. *oryzicola* is a nonvascular pathogen that enters through leaf stomata or wounds, and propagates and spreads in the intercellular spaces and the parenchyma apoplast to cause BLS in rice [19]. To reach the cell density for pathogenesis in plants, the pathogen has to be able to adapt to intercellular environments and also utilize available nutritional sources, especially carbohydrates, from the host plant. The mutation in *fbaB* does not affect the growth ability of *X. oryzae* pv. *oryzicola* when glucose, sucrose, mannose and galactose are used as the sole carbon source (data not shown), but impairs the growth rate when fructose is as the sole carbon (Figure 2), demonstrating that EMP pathway of glycolysis does not play an obvious role in sugar catabolism of *X. oryzae* pv. *oryzicola*. This is consistent with previous reports that Xanthomonads primarily employ ED, together with PPP, instead of EMP pathway of glycolysis to utilize glucose because of the lack of the phosphofructokinase (PFK) activity essential for a functional EMP, although a putative phosphofructokinase-encoding gene is annotated in the genomes of *X. oryzae* pv. *oryzae*, *X. campestris* pv. *vesicatoria*, and *X. campestris* pv. *campestris* [5], [6], [7], [13]. On the other hand, the mutagenesis in *fbaB* of *X. oryzae* pv. *oryzicola* results in the complete loss of the capability to grow when pyruvate and malate are used as the sole carbon source (Figure 2), implying that FbaB is essential for gluconeogenesis of *X. oryzae* pv. *oryzicola* (Figure 7). This is concordant with the fact that gluconeogenesis is indispensable for the pathogen to utilize pyruvate or the intermediates of the TCA cycle as the sole carbon sources in *X. campestris* pv. *campestris* [4], [13], [58]. Whether or not and how intermediates from the TCA cycle of host plants are acquired by the pathogen seems critically important in understanding mechanisms of plant-pathogen interactions.

Normally, UDP-glucose, UDP-galactose and dTDP-rhamnose are precursors or building blocks of EPS biosynthesis [7]. The galactosides UDP-glucose and UDP-galactose are synthesized from glucose-1-phosphate catalyzed from a precursor fructose-6-phosphate [7], [59]. Glucose-1-phosphate is converted from galactose, glucose or sucrose, and fructose-6-phosphate is metabolised from mannose, fructose or sucrose (Figure 7). The lack of phosphofructokinase (PFK) in xanthomonads [5], [6], [7], [13] may explain the reason that the mutation in *fbaB* of *X. oryzae* pv. *oryzicola* has no effects on EPS production when galactose, glucose, mannose and sucrose, rather than fructose, are used as the sole carbon source (data not shown). The mutagenesis in *fbaB* leads the complete loss of the ability to convert pyruvate and malate into glucose-1-phosphate via gluconeogenesis for EPS synthesis in *X. oryzae* pv. *oryzicola*, explaining the reason that the EPS production in the *fbaB* mutant is remarkably reduced when pyruvate and malate are used as the sole carbons (Table 2).

The *hrp*-encoded T3SS apparatus, together with other virulence factors, are often subject to be coordinated with the regulation of HrpG and HrpX which enables the pathogen to respond to environmental factors (such as pH and osmotic strength), plant signals (such as carbon sources, organic nitrogen, and phosphate), and catabolite repression that may be encountered during the infection [17], [18], [21], [23], [60]. This regulation is very complex and varies substantially between different *Xanthomonas*-plant pathosystems and in some cases even between closely related bacteria within the same pathosystem [61]. In this study, we found that different carbohydrates have different influences on expression of *hrpG* and *hrpX*. Sucrose, galactose, mannose, glucose, fructose and pyruvate significantly increase the expression of *hrpX* in *X. oryzae* pv. *oryzicola* when they used as the sole carbon source, while sucrose and glucose remarkably enhance the expression of *hrpG* (Figure 4). By contrast, the *hrpX* expression goes up while the *hrpG* expression decreases obviously when the pathogen grows on these carbon sources, respectively, and malate represses the expression of *hrpG* and *hrpX* (Figure 4), suggesting that other unknown factor(s) is (are) possibly involved differentially in regulation of the expression of *hrpG* and *hrpX*. This postulation conflicts with the concept that HrpG function as a positive activator upstream of HrpX in regulatory pathways of *hrp* gene expression. In fact, the expression of *hrcT* goes up when *hrpG* is mutated and there is no *hrcT* expression detected when *hrpX* is mutated in *X. oryzae* pv. *oryzicola* [20], supporting the above hypothesis.

The above findings also theoretically support the development of *hrp*-inducing media, XCV2 for *X. campestris* pv. *vesicatoria* [29], XOM2 for *X. oryzae* pv. *oryzae* [61], [62] and XOM3 for *X. oryzae* pv. *oryzicola* [25]. The major carbon source in plant leaf extract is sucrose, followed by glucose and fructose, and the dicarboxylic acid, malate, and the latter induces the secretion of extracellular enzymes and has a negative effect on the expression of the T3SS in *X. campestris* pv. campestris [63]. In *P. syringae* pv. *phaseolicola*, the expression of *hrpAB*, *hrpC*, and *hrpD* was reduced when citrate or succinate was added to fructose-or sucrose-containing medium [17]. Taken together, we assumed that hexoses from plant photosynthesis induce the expression of *hrp* genes and this action can be balanced by the intermediates from the TCA cycle of the plant pathogen. Thus, the disruption in carbon metabolic pathways reduces bacterial virulence in plants through alteration of the expression of global regulator genes, including *hrpG* and *hrpX*, in plant pathogenic bacteria. As we observed, the mutation in *fbaB* of *X. oryzae* pv. *oryzicola* leads the dysfunction of gluconeogenesis and the accumulation of intermediates, like malate, from the TCA cycle represses the expression of *hrpG* and *hrpX* (Figure 4 and 5B), enhances the bacterium to use the TCA intermediates by the help of HrcC, HrpE and Hpa3 (Figure 5B, 6 and 7), and may also affect the ability of the organism to obtain nutritents from the environment.

The interesting finding in this report is that the promoter region of *fbaB* of *X. oryzae* pv. *oryzocola* assembles the *cis*-element of PIP-box which is taken as the sequence of HrpX regulons (Figure 3A). The highly conserved PIP-box of the *fbaB* homologue in other typical *Xanthomonas* species (data not shown) suggests that the expression of *fbaB* may commonly be negatively regulated by HrpG and HrpX. Protein secretion assays demonstrated that FbaB is not secreted through the T2SS and T3SS (data not shown). In fact, the expression level of *fbaB* in the *hrpG* mutant is higher than than in the *hrpX* mutant (Figure 3B), suggesting yet unknown factor(s) may strongerly regulate the expression of HrpX than HrpG. This is consistent with the following findings. The base substitution in the fifth residue of the left motif TTCGT of the PIP-box significantly reduced the expression of *fbaB* when *hrpG* was mutated rather than in the *hrpX* mutant (Figure 3C). However, the substitution in the right motif TTCGT led the expression of *fbaB* to be 15–20 fold higher than the wild-type promoter when *hrpG* and *hrpX* are disrupted, respectively and the promoter activity in the *hrpX* mutant is significantly higher than that in the *hrpG* mutant (Figure 3C), implying that the alteration of the binding sites of the *fbaB* PIP-box promoter makes the expression of *fbaB* released from the regulation of HrpG and HrpX together with a yet unknown regulator (Figure 7). This unknown regulator may activate the expression of *fbaB* which is also regulated by the HrpG and HrpX cascade (Figure 7), or regulate the expression of HrpG by phosphorylation as speculated in *R. solanacearum* [64]. Unfortunately, our electrophoretic mobility shift assay (EMSA) showed that HrpX did not bind the *fbaB* promoter (data not shown), suggesting that HrpX, a transcriptional activator, may form a complex with a yet unknown factor to regulate the expression of *fbaB*. The expression of this unknown factor may be inhibited by intermediates, like malate, from the TCA cycle in *X. oryzae* pv. *oryzicola*, resulting in the lower expression of HrpG and HrpX when the block of gluconeogenesis is made by the *fbaB* mutation (Figure 3, 4, 5 and 6).

In addition, the expression of *hrcC*, *hrpE* and *hpa3*, other than other *hrp*-*hrc*-*hpa* genes, of *X. oryzae* pv. *oryzicola* is still activated when *fbaB* is mutated. Previously, we found that the mutation of *hrpG* and *hrpX* does not abolish the expression of *hrcC*, *hrpE* and *hpa3* and postulated that a yet unknown factor may influence the expression of these genes [20], [53]. This speculation is in accordance with our hypothesis in this report that the hexoses from plant photosynthesis induce the expression of virulence-related genes, including *hrp* and *fbaB* genes, and the intermediates, like malate, from the TCA cycle of *X. oryzae* pv. *oryzicola* repress the expression of the unknown factor gene that will in turn, directly or indirectly, suppress the expression of HrpG and HrpX and increase the expression of *hrcC*, *hrpE* and *hpa3* which is involved in nutrient acquirement of pyruvate and malate when *X. oryzae* pv. *oryzicola* contacts the host cells (Figure 5 and 6). Importantly, this report may also provide clues to investigate a yet unknown factor which presumably plays a central role in regulation between carbohydrate metabolism and the *hrp* system of *X. oryzae* pv. *oryzicola* mediated by pyruvate and malate (Figure 7).

## Supporting Information

Figure S1 **Schematic map and molecular analysis of** ***fabB*** **mutation in** ***X. oryzae*** **pv.** ***oryzicola*** **.** The positions and orientations of *Xoryp_17640*, encoding FbaB, and other adjacent ORFs are shown by using the genome sequence of *X. oryzae* pv. *oryzicola* BLS256 strain as the reference (http://cmr.jcvi.org/cgi-bin/CMR/GenomePage.cgi?org=Xoc). Arrows indicate locations and orientations of the ORFs or protein IDs, and lines indicate the intergenic sequences. ▾above ORF *Xoryp_17640* presents the insertion site of a transposon Tn5 derivative in mutant Mxoc0504. A non-polar construction of a *fabB* deletion mutant was sketched (see Materials and methods for detail). The white box stands for a 349 bp deletion of *fbaB*. The *fbaB* mutant was verified by PCR with the primer pair upF/downR (Table S1) and by Southern hybridization with a 546 bp fragment of *fbaB* gene as the probe. Lane 1, the wild-type strain RS105; Lane2, the *fabB* mutant RΔ*fabB*; Lane M, DL2000 or λ - *Eco*T14 DNA marker (TaKaRa, Dalian, China).
(TIFF)

Table S1 **Primers used in this study.**
(DOC)

## References

[1] Eisenreich W, Dandekar T, Heesemann J, Goebel W (2010). Carbon metabolism of intracellular bacterial pathogens and possible links to virulence.. Nat Rev Microbiol.

[2] Tamir-Ariel D, Navon N, Burdman S (2007). Identification of genes in *Xanthomonas campestris* pv. *vesicatoria* induced during its interaction with tomato.. J Bacteriol.

[3] Wang LF, Rong W, He CZ (2008). Two Xanthomonas extracellular polygalacturonases, PghAxc and PghBxc, are regulated by type III secretion regulators HrpX and HrpG and are required for virulence.. Mol Plant-Microbe Interact.

[4] Tang DJ, He YQ, Feng JX, He BR, Jiang BL (2005). *Xanthomonas campestris* pv. *campestris* possesses a single gluconeogenic pathway that is required for virulence.. J Bacteriol.

[5] Zagallo AC, Wang CH (1967). Comparative Glucose Catabolism of *Xanthomonas* Species.. J Bacteriol.

[6] Kim SY, Lee BM, Cho JY (2010). Relationship between glucose catabolism and xanthan production in *Xanthomonas oryzae* pv. *oryzae*.. Biotechnol Lett.

[7] Letisse F, Chevallereau P, Simon JL, Lindley N (2002). The influence of metabolic network structures and energy requirements on xanthan gum yields.. J Biotechnol.

[8] Lee BM, Park YJ, Park DS, Kang HW, Kim JG (2005). The genome sequence of *Xanthomonas oryzae* pathovar *oryzae* KACC10331, the bacterial blight pathogen of rice.. Nucleic Acids Res.

[9] Salzberg SL, Sommer DD, Schatz MC, Phillippy AM, Rabinowicz PD (2008). Genome sequence and rapid evolution of the rice pathogen *Xanthomonas oryzae* pv. *oryzae* PXO99^A^.. BMC Genomics.

[10] Qian W, Jia Y, Ren SX, He YQ, Feng JX (2005). Comparative and functional genomic analyses of the pathogenicity of phytopathogen *Xanthomonas campestris* pv. *campestris*.. Genome Res.

[11] da Silva AC, Ferro JA, Reinach FC, Farah CS, Furlan LR (2002). Comparison of the genomes of two Xanthomonas pathogens with differing host specificities.. Nature.

[12] Thieme F, Koebnik R, Bekel T, Berger C, Boch J (2005). Insights into genome plasticity and pathogenicity of the plant pathogenic bacterium *Xanthomonas campestris* pv. *vesicatoria* revealed by the complete genome sequence.. J Bacteriol.

[13] Lu GT, Xie JR, Chen L, Hu JR, An SQ (2009). Glyceraldehyde-3-phosphate dehydrogenase of *Xanthomonas campestris* pv. *campestris* is required for extracellular polysaccharide production and full virulence.. Microbiology.

[14] Mellgren EM, Kloek AP, Kunkel BN (2009). Mqo, a tricarboxylic acid cycle enzyme, is required for virulence of *Pseudomonas syringae* pv. *tomato* strain DC3000 on Arabidopsis thaliana.. J Bacteriol.

[15] Aldon D, Brito B, Boucher C, Genin S (2000). A bacterial sensor of plant cell contact controls the transcriptional induction of *Ralstonia solanacearum* pathogenicity genes.. EMBO J.

[16] Brito B, Aldon D, Barberis P, Boucher C, Genin S (2002). A signal transfer system through three compartments transduces the plant cell contact-dependent signal controlling *Ralstonia solanacearum hrp* genes.. Mol Plant-Microbe Interact.

[17] Rahme LG, Mindrinos MN, Panopoulos NJ (1992). Plant and environmental sensory signals control the expression of *hrp* genes in *Pseudomonas syringae* pv. phaseolicola.. J Bacteriol.

[18] Schulte R, Bonas U (1992). Expression of the *Xanthomonas campestris* pv. *vesicatoria hrp* gene cluster, which determines pathogenicity and hypersensitivity on pepper and tomato, is plant inducible.. J Bacteriol.

[19] Zou LF, Wang XP, Xiang Y, Zhang B, Li YR (2006). Elucidation of the *hrp* Clusters of *Xanthomonas oryzae* pv. *oryzicola* That Control the Hypersensitive Response in Nonhost Tobacco and Pathogenicity in Susceptible Host Rice.. Appl Environ Microbiol.

[20] Li YR, Zou HS, Che YZ, Cui YP, Guo W (2011). A novel regulatory role of HrpD6 in regulating *hrp-hrc-hpa* genes in *Xanthomonas oryzae* pv. *oryzicola*.. Mol Plant-Microbe Interact.

[21] Ankenbauer RG, Nester EW (1990). Sugar-mediated induction of Agrobacterium tumefaciens virulence genes: structural specificity and activities of monosaccharides.. J Bacteriol.

[22] Tamir-Ariel D, Rosenberg T, Burdman S (2011). The *Xanthomonas campestris* pv. *vesicatoria citH* gene is expressed early in the infection process of tomato and is positively regulated by the TctDE two-component regulatory system.. Mol Plant Pathol.

[23] Tung SY, Kuo TT (1999). Requirement for phosphoglucose isomerase of *Xanthomonas campestris* in pathogenesis of citrus canker.. Appl Environ Microbiol.

[24] Guo XX, Zou HS, Li YR, Zou LF, Chen GY (2010). *hrpD6* gene determines 27 *Xanthomonas oryzae* pv. *oryzae* to trigger hypersensitive response in tobacco and pathogenicity in rice.. Acta Microbiologica Sinica.

[25] Xiao YL, Li YR, Liu ZY, Xiang Y, Chen GY (2007). Establishment of the *hrp*-inducing systems for the expression of the *hrp* genes of *Xanthomonas oryzae* pv. *oryzicola*.. Acta Microbiologica Sinica.

[26] Cunnac S, Boucher C, Genin S (2004). Characterization of the cisacting regulatory element controlling HrpB-mediated activation of the type III secretion system and effector genes in *Ralstonia solanacearum*.. J Bacteriol.

[27] He SY, Nomura K, Whittam TS (2004). Type III protein secretion mechanism in mammalian and plant pathogens.. Biochim Biophys Acta.

[28] Hueck CJ (1998). Type III protein secretion systems in bacterial pathogens of animals and 16 plants.. Microbiol Mol Biol Rev.

[29] Weber E, Koebnik R (2005). Domain Structure of HrpE, the Hrp Pilus Subunit of 11 *Xanthomonas campestris* pv. *vesicatoria*.. J Bacteriol.

[30] Büttner D, Bonas U (2002a). Port of entry-the type III secretion translocon.. Trends in Microbiol.

[31] Büttner D, Bonas U (2002b). Getting across-bacterial type III effector proteins on their 16 way to the plant cell.. EMBO J.

[32] Büttner D, Nennstiel D, Klusener B, Bonus U (2002). Functional analysis of HrpF, 21 a putative type III translocon protein from *X. campestris* pv. *vesicatoria*.. J Bacteriol.

[33] Sugio A, Yang B, White FF (2005). Characterization of the *hrpF* Pathogenicity 27 Peninsula of *Xanthomonas oryzae* pv. *oryzae*.. Mol Plant-Microbe Interact.

[34] Wengelnik K, Rossier O, Bonas U (1999). Mutations in the regulatory gene *hrpG* of *Xanthomonas campestris* pv. *vesicatoria* result in constitutive expression of all *hrp* genes.. J Bacteriol.

[35] Wengelnik K, Van den Ackerveken G, Bonas U (1996). HrpG, a key *hrp* regulatory protein of *Xanthomonas campestris* pv. *vesicatoria* is homologous to two-component response regulators.. Mol Plant-Microbe Interact.

[36] Wengelnik K, Bonas U (1996). HrpXv, an AraC-type regulator, activates expression of five of the six loci in the *hrp* cluster of *Xanthomonas campestris* pv. *vesicatoria*.. J Bacteriol.

[37] Furutani A, Nakayama T, Ochiai H, Kaku H, Kubo Y (2006). Identification of novel HrpXo regulons preceded by two cis-acting elements, a plant-inducible promoter box and a -10 box-like sequence, from the genome database of *Xanthomonas oryzae* pv. *oryzae*.. FEMS Microbiol Lett.

[38] Koebnik R, Krüger A, Thieme F, Urban A, Bonas U (2006). Specific binding of the *Xanthomonas campestris* pv. *vesicatoria* AraC-type transcriptional activator HrpX to plant-inducible promoter boxes.. J Bacteriol.

[39] Tsuge S, Terashima S, Furutani A, Ochiai H, Oku T (2005). Effects on promoter activity of base substitutions in the cis-acting regulatory element of HrpXo regulons in *Xanthomonas oryzae* pv. *oryzae*.. J Bacteriol.

[40] Huang DL, Tang DJ, Liao Q, Li XQ, He YQ (2009). The Zur of *Xanthomonas campestris* is involved in hypersensitive response and positively regulates the expression of the *hrp* cluster via *hrpX* but not *hrpG*.. Mol Plant-Microbe Interact.

[41] Noël L, Thieme F, Nennstiel D, Bonas U (2002). Two novel type III system-secreted proteins of *Xanthomonas campestris* pv. *vesicatoria* are encoded within the hrp pathogenicity island.. J Bacteriol.

[42] Oku T, Tanaka K, Iwamoto M, Inoue Y, Ochiai H (2004). Structural conservation of *hrp* gene cluster in *Xanthomonas oryzae* pv. *oryzae*.. J Gen Plant Pathol.

[43] Staslawicz BJ, Mudgett MB, Dangl JL, Galan JE (2001). Common and contrasting themes of plant and animal diseases.. Science.

[44] Tsuge S, Nakayama T, Terashima S, Ochiai H, Furutani A (2006). Gene involved in transcriptional activation of the *hrp* regulatory gene *hrpG* in *Xanthomonas oryzae* pv. *oryzae*.. J Bacteriol.

[45] He YW, Ng AY, Xu M, Lin K, Wang LH (2007). *Xanthomonas campestris* cell-cell communication involves a putative nucleotide receptor protein Clp and a hierarchical signalling network.. Mol Microbiol.

[46] Islam MR, Kabir MS, Hirata H, Tsuge S, Tsuyumu S (2009). A leucine-rich protein, LrpX, is a new regulator of *hrp* genes in *Xanthomonas oryzae* pv. *oryzae*.. J Gen Plant Pathol.

[47] Zhang SS, He YQ, Xu LM, Chen BW, Jiang BL (2008). A putative *colR* (*XC1049*)-*colS* (*XC1050*) two-component signal transduction system in *Xanthomonas campestris* positively regulates *hrpC* and *hrpE* operons and is involved in virulence, the hypersensitive response and tolerance to various stresses.. Res Microbiol.

[48] Lee SW, Jeong KS, Han SW, Lee SE, Phee BK (2008). The *Xanthomonas oryzae* pv. *oryzae* PhoPQ two-component system is required for AvrXA21 activity, *hrpG* expression, and virulence.. J Bacteriol.

[49] Zou HS, Yuan L, Guo W, Li YR, Che YZ (2011). Construction of a Tn5-Tagged Mutant Library of *Xanthomonas oryzae* pv. *oryzicola* as An Invaluable Resource for Functional Genomics.. Curr Microbiol.

[50] Miller JH (1972). Experiments in molecular genetics.

[51] Sambrook J, Fritsch EF, Maniatis T (1989). Molecular Cloning: A Laboratory Manual..

[52] Turner PE (2004). Phenotypic plasticity in bacterial plasmids.. Genetics.

[53] Jiang J, Zou HS, Li YR, Chen GY (2009). Expression of the *hrcC*, *hrpE* and *hpa3* genes is not regulated by the *hrpG* and *hrpX* genes in a rice pathogen *Xanthomonas oryzae* pv. *oryzicola*.. Wei Sheng Wu Xue Bao.

[54] Wang L, Makino S, Subedee A, Bogdanove AJ (2007). Novel candidate virulence factors in rice pathogen *Xanthomonas oryzae* pv. *oryzicola* as revealed by mutational analysis.. Appl Environ Microbiol.

[55] Tang JL, Liu YN, Barber CE, Dow JM, Wootton JC (1991). Genetic and molecular analysis of a cluster of *rpf* genes involved in positive regulation of synthesis of extracellular enzymes and polysaccharide in *Xanthomonas campestris* pathovar *campestris*.. Mol Gen Genet.

[56] Denny TP (1995). Involvement of bacterial polysaccharides in plant pathogenesis.. Annu Rev Phytopathol.

[57] Yang W, Liu Y, Chen L, Gao T, Hu B (2007). Zinc uptake regulator (*zur*) gene involved in zinc homeostasis and virulence of *Xanthomonas oryzae* pv. *oryzae* in rice.. Curr Microbiol.

[58] Say RF, Fuchs G (2010). Fructose 1,6-bisphosphate aldolase/phosphatase may be an ancestral gluconeogenic enzyme.. Nature.

[59] Hochster RM, Katznelson H (1958). On the mechanism of glucose-6-phosphate oxidation in cell-free extracts of *Xanthomonas phaseoli* (XP8).. Can J Biochem Physiol.

[60] Winans SC (1990). Transcriptional induction of an Agrobacterium regulatory gene at tandem promoters by plant-released phenolic compounds, phosphate starvation, and acidic growth media.. J Bacteriol.

[61] Seo YS, Sriariyanun M, Wang L, Pfeiff J, Phetsom J (2008). A two-genome microarray for the rice pathogens *Xanthomonas oryzae* pv. oryzsae and *X. oryzae* pv. *oryzicola* and its use in the discovery of a difference in their regulation of *hrp* genes.. BMC Microbiol.

[62] Furutani A, Tsuge S, Ohnishi K, Hikichi Y, Oku T (2004). Evidence for HrpXo-dependent expression of type II secretory proteins in *Xanthomonas oryzae* pv. *oryzae*.. J Bacteriol.

[63] Watt TF, Vucur M, Baumgarth B, Watt SA, Niehaus K (2009). Low molecular weight plant extract induces metabolic changes and the secretion of extracellular enzymes, but has a negative effect on the expression of the type-III secretion system in *Xanthomonas campestris* pv. *campestris*.. J Biotechnol.

[64] Yoshimochi T, Hikichi Y, Kiba A, Ohnishi K (2009). The global virulence regulator PhcA negatively controls the *Ralstonia solanacearum hrp* regulatory cascade by repressing expression of the PrhIR signaling proteins.. J Bacteriol.

[65] Wang YP, Zou LF, Zhou D, Chen GY (2009). Key roles of *hrpE* gene of *Xanthomonas oryzae* pv. *oryzicola* in formation of Hrp pilus and pathogenicity in rice.. Acta Phytopathologica Sinica.

