# Supplementary figures and images for: Fructose-Bisphophate Aldolase Exhibits Functional Roles between Carbon Metabolism and the hrp System in Rice Pathogen Xanthomonas oryzae pv. oryzicola
Source: PLoS One. 2012 Feb 22;7(2):e31855. doi: 10.1371/journal.pone.0031855 (PMC3285194; doi:10.1371/journal.pone.0031855)

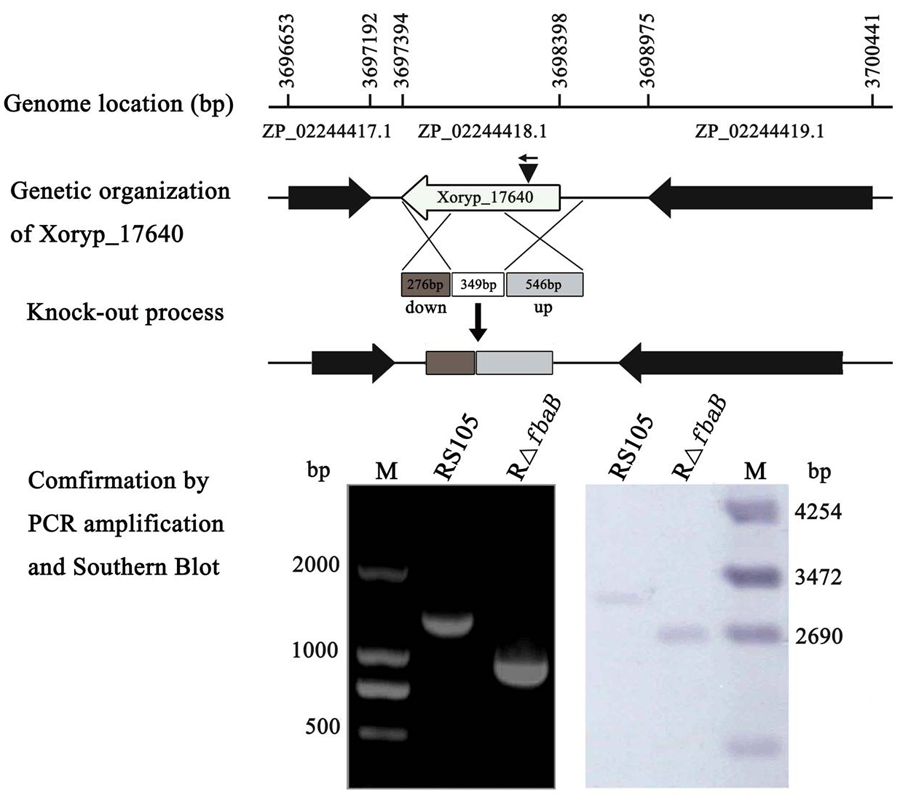

Supplement: Figure S1 — Schematic map and molecular analysis of fabB mutation in X. oryzae pv. oryzicola . The positions and orientations of Xoryp_17640, encoding FbaB, and other adjacent ORFs are shown by using the genome sequence of X. oryzae pv. oryzicola BLS256 strain as the reference (http://cmr.jcvi.org/cgi-bin/CMR/GenomePage.cgi?org=Xoc). Arrows indicate locations and orientations of the ORFs or protein IDs, and lines indicate the intergenic sequences. ▾above ORF Xoryp_17640 presents the insertion site of a transposon Tn5 derivative in mutant Mxoc0504. A non-polar construction of a fabB deletion mutant was sketched (see Materials and methods for detail). The white box stands for a 349 bp deletion of fbaB. The fbaB mutant was verified by PCR with the primer pair upF/downR (Table S1) and by Southern hybridization with a 546 bp fragment of fbaB gene as the probe. Lane 1, the wild-type strain RS105; Lane2, the fabB mutant RΔfabB; Lane M, DL2000 or λ - EcoT14 DNA marker (TaKaRa, Dalian, China). (TIFF) [file pone.0031855.s001.tiff]
