# Supplementary material for: Fructose-Bisphophate Aldolase Exhibits Functional Roles between Carbon Metabolism and the hrp System in Rice Pathogen Xanthomonas oryzae pv. oryzicola
Source: PLoS One. 2012 Feb 22;7(2):e31855. doi: 10.1371/journal.pone.0031855 (PMC3285194; doi:10.1371/journal.pone.0031855)
Supplement: Table S1 — Primers used in this study. (DOC) [file pone.0031855.s002.doc]

**Table S1 Primers used in this study**

| **Purpose** | **Primes** | **Sequence (5’→3’; restriction sites underlined)** | **Description** |
| --- | --- | --- | --- |
| Mutagenesis | fbaBI-F/  fbaBI-R | TGAGGATCCATGCTCAGCTCTGCCGATTCC/  ATAGAATTCTACTCCTCCAGACGTGCACGC | A 546 bp fragment left to *fbaB* |
|  | fbaBII-F/  fbaBII-R | ATAGAATTCATGTGCCTGAAGTCGACCGTG/  ATTCTGCAGTCAACCCTTCCACTTACCCAG | A 276 bp fragment right to *fbaB* |
| PCR  verification | fbaBI-F/  fbaBII-R | TGAGGATCCATGCTCAGCTCTGCCGATTCC/  ATTCTGCAGTCAACCCTTCCACTTACCCAG | A 1171 bp portion consisting of the left and right fragments above |
| Southern Blot | fbaBI-F/  fbaBI-R | TGAGGATCCATGCTCAGCTCTGCCGATTCC/  ATAGAATTCTACTCCTCCAGACGTGCACGC | A 546 bp fragment of *fbaB* was used as a probe for southern blot |
| Complemented construction | fbaB*-*F/  fbaB*-*R | ATAAAGCTTGAAGTGGCGAAGTGGCAAGAG/  ATAGGTACCTCAACCCTTCCACTTACCCAG | A 1302 bp fragment containing the entire *fbaB* gene and its own promoter |
| RT-PCR | 16S rRNA*-*F/  16S rRNA*-*R | AATGGGCGCAAGCCTGATC/  TTTGTCACCGGCGGTCTCC | A 801 bp DNA of *16S rRNA* gene |
|  | hrcC-F/  hrcC-R | GCTGGAGGTAATGACCGGAAT/  ATGCAGAGCTATGGCACCCT | A 567 portion of *hrcC* |
|  | hrpE-F/  hrpE-R | CGCTTCGAACAGGGTATGGAT/  GCCTTGCCGATGAACTTGTT | A 211 bp fragment of *hrpE* |
|  | hrpD5-F/  hrpD5-R | GCAGGCGCACGATTAGATTT/  CAATGCCTGGGTGATAGCGA | A 571 bp portion of *hrpD5* |
|  | hpa3-F/  hpa3-R | TAGTGCAGCAGCCACCTCTTCA/  AGCAAATCCGGATTACCACCA | A 357 bp fragment of *hpa3* |
|  | hrpG-F/  hrpG-R | ATCTTTGTCTTGCAGCGCGA/  TTTCGACTGCGCCAATGACAT | A 455 bp of *hrpG* |
|  | hrpX-F/  hrpX-R | AAGAAGAGGCCGAAGACGCGTA/  GGCGGAAATATCGTCGGAGA | A 893 bp of *hrpX* |
|  | hpa1-F/  hpa1-R | TTCCAGGTTGACCAAAGCCA/  AGAAGCTGCTGGCGAAACTG | A 346 bp of *hpa1* |
|  | hrpB1-F/  hrpB1-R | GAACTTCCATAGGTGCTGACGATT/  CGCCAGCGACTTCCGATATT | A 534 bp of *hrpB1* |
|  | hrcU-F/  hrcU-R | AGAAGCCGACCGAGAAGAAACT/  CGCCCTCGCTTTCCTTGTATT | A 675 bp of *hrcU* |
|  | hrpD6-F/  hrpD6-R | CGATGCGGTCACTCAGGATAT/  CTGGCGATATGCACGACGAT | A 216 bp of *hrpD6* |
|  | hrpF*-*F/  hrpF*-*R | TGTTCTTTGCGATCGGTTCTCA/  GCAGATAGTCGGAATAGCGATACA | A 588 bp of *hrpF* |
| Reporter construction | gusA*-*F/  gusA*-*R | ATAGGTACCATGTTACGTCCTGTAGAAACC/  ATAAAGCTTTCATTGTTTGCCTCCCTGCTG | a 1812 bp *gusA* gene |
|  | fbaBa*-*F/  fbaBa*-*R | ATAGAATTCGAAGTGGCGAAGTGGCAAGAG/  ATAGGTACCGATGGCGATGATGCCCTTACC | A 366 bp promoter of *fbaB* |
|  | fbaBa-F/  fbaBb-R | ATGAATTCGAAGTGGCGAAGTGGCAAGAG/  TAGGTACCTTCGATGCTCATGTAGTTCCTGTGGCAGCTGCGGGGGAGAACGAAAGTATAGC GGCGAA | The first TTCGT of the PIP-box was replaced by TTCGC in *fbaB* promoter |
|  | fbaBa-F/  fbaBc-R | ATGAATTCGAAGTGGCGAAGTGGCAAGAG/  TAGGTACCTTCGATGCTCATGTAGTTCCTGTGGCAGCTGCGGGGGAGAGCGAA | The second TTCGT of the PIP-box was replaced by TTCGC in *fbaB* promoter |
|  | fbaBa-F/  fbaBd-R | ATGAATTCGAAGTGGCGAAGTGGCAAGAG/  TAGGTACCTTCGATGCTCATGTAGTTCCTGTGGCAGCTGCGGGGGAGAACGAAAGTATAGCGATACA | The first TTCGT of the PIP-box was mutated to TGATA in *fbaB* promoter |
| Real-time quantitative  RT-PCR | 16S rRNA*-*F/  16S rRNA*-*R | AATGGGCGCAAGCCTGATC/  AACCACCACCTACGCACGC | *16S rRNA* gene fragment, 221 bp |
|  | gusA-F/  gusA*-*R | TAACTATGCCGGGATCCATCG/  AGTCCCGCTGGTGCCTTGTCC | *gus* gene fragment,  205 bp |
|  | fbaB*-*F/  fbaB*-*R | AAGTGGCGTGCGGTGATCAAC/  GTCGCTTCGGTGACTTCGTAG | A 188 bp fragment of *fbaB* |
|  | hrcC-F/  hrcC-R | GGATCTCGCCAGAGGTGACCG/  GAAAGCGCAAATCGTCCAGGC | *hrcC* gene fragment,  231 bp |
|  | hrpE-F/  hrpE-R | CTTCGAACAGGGTATGGATGG/  TTGAGCTGCGTGATCTTGTTG | *hrpE* gene fragment,  162 bp |
|  | hrpD5-F/  hrpD5-R | GCAGGCGCACGATTAGATTTG/  CGGCACCAGCATCACCAACAC | *hrpD5* gene fragment,  223 bp |
|  | hpa3-F/  hpa3-R | TTGGGTTACCTGGTGAGCTTG/  CCAGCCATACTCGGTCAGCAG | *hpa3* gene fragment,  215 bp |
|  | hrpG-F/  hrpG-R | GTTGCTCCGCGACGAAAATAC/  CTTGCGCAGCTTGTAGATATG | *hrpG* gene fragment,  199 bp |
|  | hrpX-F/  hrpX-R | GGCGATTGTTGTCTTTTGCTC/  GACCTCATCGTCGGCTCCATC | *hrpX* gene fragment,  209 bp |
|  | hpa1-F/  hpa1-R | GGTTGACCAAAGCCAGAACGC/  CATTCTCGCCACCCTGCTGAC | *hpa1* gene fragment,  167 bp |
|  | hrpB1-F/  hrpB1-R | GAAGATTCAATGTCCCGGGTC/  ACGTAATTGCCGCGCTTGATC | *hrpB1* gene fragment,  180 bp |
|  | hrcU-F/  hrcU-R | GAGACACCGCTTGATATTGCC/  GCCCTCGCTTTCCTTGTATTC | *hrcU* gene fragment,  180 bp |
|  | hrpD6-F/  hrpD6-R | AGATTCGCGGGCACTGGTCTG/  CTGGCGATATGCACGACGATG | *hrpD6* gene fragment,  109 bp |
|  | hrpF*-*F/  hrpF*-*R | TTGAAGGAAGCGCTGAAAGGA/  AAGTCTGTGAGGTCCTGCGCC | *hrpF* gene fragment,  113 bp |

N Presents the subsituted bases.
